# COVID-19, Healthcare Workers and Mental Wellbeing: Lessons From One Very Cold and Another Very Hot Part of the World

**DOI:** 10.1177/07067437221094341

**Published:** 2022-04-21

**Authors:** Danilo Arnone, Reshma Ramaraj, Emmanuel Stip

**Affiliations:** 1 Department of Psychiatry and Behavioural Science, College of Medicine and Health Sciences, 62776 United Arab Emirates University, Al Ain, United Arab Emirates; 2 Centre for Affective Disorders, Department of Psychological Medicine, Institute of Psychiatry, Psychology and Neuroscience, King’s College London, London, UK; 3 Centre de Recherche Institut Universitaire en Santé 12368 Mentale de Montréal, Université de Montréal, Montréal, QC, Canada

Dufour and colleagues' work investigated the frequency of psychological distress among healthcare workers (HW) in the province of Quebec during and after the first wave of COVID-19 pandemic.^1^ Authors reported peak point prevalence of mental health symptoms in HW of 22.2% for post-traumatic stress, 29.9% for depression and 26.9% for anxiety, consistent with similar data collected around the World.^2^ These findings are mirrored in a recent cross-sectional survey conducted in Abu Dhabi in the United Arab Emirates (UAE) at the zenith of the first wave of the pandemic, suggesting that an overall 39% of HW were affected by symptoms of depression, anxiety and stress, 13% of them severely.^3^ An email with a link to an online questionnaire was sent out to all UAE’s largest public health sector employees, a total of 18,371. A total of 2184 responses were collected. The questionnaire consisted of the Depression and Anxiety Screening Scale (DASS-21) and basic demographic questions. The study protocol also included a dedicated psychological support helpline for staff members. However, this study on a self-selected sample of HW was a telephone-based helpline for HW, and it is not fully comparable.

UAE has a similar gross national income and population size than Quebec. Abu Dhabi is the capital and the largest emirate. The healthcare system is decentralised like Canada albeit primarily insurance based rather than publicly funded, offering variable degrees of coverage. At times of HW crisis (such as a pandemic) it is imperative to ensure that HW have rapid and effective access to health care including available psychological support, an issue actively discussed in Quebec.^4^

Abu Dhabi healthcare services offer free psychological support to HW by virtue of a telephone-based helpline. Data from this service during the first wave suggest that 23% of contacts were related to COVID-19. Main reasons for contact primarily included COVID-19 work stress, hazardous work practices, exposure to death-related traumatic experiences, loneliness and insecurity, family separation, self-isolation (also pertaining to the inability to travel) and job insecurity.^3^ In both studies respondents were more frequently women.^1,3^

Dufour and colleagues utilised a mobile application which incorporated three main mental health wellbeing indicators to understand the evolution of psychological distress over time. The longitudinal analysis of this data indicated that a small number of individuals (∼8%) experienced a delayed onset. This suggests that the impact of contributing factors fluctuates in relation to virulence and counteractive measures. The authors also identified sub-chronic symptoms (∼7%) supporting the notion of the existence of possible fluctuating triggers and maintaining factors which could be addressed for those at risk. The authors finally concluded that mental health resilience is the predominant response for most HW facing the challenge of COVID-19 pandemic (∼67%) and that a large number of affected individuals fully recover (∼19%).

It is important to identify those at risk over time as vulnerability fluctuates. Helplines and mobile applications could potentially identify risks and maintaining factors, detect psychopathology and offer cost-effective practical interventions aiming at harm prevention and minimisation.^5^ These interventions could include individualized emotional support (online counselling support, video chats, online forums and support groups, access to psychiatric and psychological services). Findings from diverse research conducted in different parts of the World offers opportunities to identify mental health needs of HW at critical times.
